# Risk of adverse pregnancy and perinatal outcomes after high technology infertility treatment: a comprehensive systematic review

**DOI:** 10.1186/s12958-016-0211-8

**Published:** 2016-11-04

**Authors:** Stefano Palomba, Roy Homburg, Susanna Santagni, Giovanni Battista La Sala, Raoul Orvieto

**Affiliations:** 1 Center of Reproductive Medicine and Surgery, Arcispedale Santa Maria Nuova (ASMN)-Istituto di Ricovero e Cura a Carattere Scientifico (IRCCS), Viale Risorgimento 80, 42123 Reggio Emilia, Italy; 2 Homerton Fertility Unit, Homerton University Hospital, Homerton Row, London, UK; 3 University of Modena, Reggio Emilia, Italy; 4 Department of Obstetrics and Gynecology, Chaim Sheba Medical Center (Tel Hashomer), Ramat Gan, Israel; 5 Sackler Faculty of Medicine, Tel Aviv University, Tel Aviv, Israel

**Keywords:** ART, Complications, Infertility, Obstetric, Pregnancy, Subfertility

## Abstract

In the literature, there is growing evidence that subfertile patients who conceived after infertility treatments have an increased risk of pregnancy and perinatal complications and this is particularly true for patients who conceived through use of high technology infertility treatments. Moreover, high technology infertility treatments include many concomitant clinical and biological risk factors. This review aims to summarize in a systematic fashion the current evidence regarding the relative effect of the different procedures for high technology infertility treatments on the risk of adverse pregnancy and perinatal outcome. A literature search up to August 2016 was performed in IBSS, SocINDEX, Institute for Scientific Information, PubMed, Web of Science and Google Scholar and an evidence-based hierarchy was used to determine which articles to include and analyze. Data on prepregnancy maternal factors, low technology interventions, specific procedures for male factor, ovarian tissue/ovary and uterus transplantation, and chromosomal abnormalities and malformations of the offspring were excluded. The available evidences were analyzed assessing the level and the quality of evidence according to the Oxford Centre for Evidence-Based Medicine guidelines and the Grading of Recommendations Assessment, Development, and Evaluation system, respectively. Current review highlights that every single procedure of high technology infertility treatments can play a crucial role in increasing the risk of pregnancy and perinatal complications. Due to the suboptimal level and quality of the current evidence, further well-designed studies are needed.

## Background

Throughout the years, it has always been clear to scientists that the primary endpoint in reproductive medicine was the healthy baby and that all other endpoints would be considered only a surrogate [1, 2]. The published infertility clinical trials have rarely reported clear data about the possible harm of the medical, surgical and biological procedures for enhancing fertility [3, 4], as well as giving very little relevance to long-term effects of those procedures on maternal and offspring health. Only 4.8 % and 5.7 % of randomized controlled trials (RCTs) on infertility treatments reported on perinatal and maternal outcome [5]. This is probably because obstetrical and infant care are delivered by other providers and patients are lost to follow-up.

Notwithstanding these limitations, more and more data available in the literature seem to demonstrate that pregnancy following infertility treatments are at higher risk of adverse pregnancy and perinatal outcomes when compared with those after natural conception (NC) independently from scientific approach [6], and this is particularly true for pregnancies achieved thanks to high technology infertility treatments [5]. The majority of this risk is a “pure” iatrogenic risk due to the high rates of multiple births, i.e. 41.1 % of the United States (US) infants conceived with assisted reproductive technologies (ART) were born as multiple-birth infants compared with only 3.5 % of infants among the general birth population [7]. The rate of multiple deliveries following ART represents about 18.7 % of total multiple-birth infants [7]. However, because also singleton infants conceived with ART are at higher risk of preterm birth (PTB) and low birthweight (LBW) [7], other determinants cannot be excluded. Patients’ characteristics including the infertility state [8, 9] and many preconception risk factors for subfertility [10–12] can largely increase the absolute and relative risk of obstetric morbidity.

Although systematic reviews with and/or without meta-analysis have been published on specific topics, at the moment no comprehensive review is available in the literature, discussing the impact on maternal and perinatal outcome of each element and/or clinical/biological choice which comprise the high technology infertility treatments. Based on these considerations, the aim of the current document was to comprehensively review in a systematic fashion the hitherto published evidences regarding the effects of high technology infertility treatments on the obstetric risk of patients with female and couple infertility. The effects on the risk of chromosomal abnormalities and malformations of the offspring was not a study aim.

## Materials and methods

The methodology used for the current systematic review consisted of searching all available articles for each specific issue to explore the relationship between high technology infertility treatments and pregnancy and perinatal complications. High technology infertility treatments were considered ﻿as﻿ all interventions for fertility enhancement including manipulation of female gametes. The Preferred Reporting Items for Systematic Reviews and Meta-Analyses (PRISMA) Statement [13] was followed but after comprehensive search all the authors agreed to prepare the document in a narrative fashion in consideration of the multifaceted aspects to discuss.

Multiple strategies were used to search and identify relevant demographic, epidemiological, clinical and experimental studies. Sociological online libraries (IBSS, SocINDEX), Institute for Scientific Information, PubMed, Web of Science and Google Scholar were consulted. Only articles written in English were considered. The search was conducted independently by two authors (S.P. and S.S.). The literature available up to August 2016 was captured, including all available studies which reported data about the relationship between each fertility technique and the related obstetric and perinatal complications, matching every intervention with every potential obstetric disorder and perinatal health impact, as shown in Table 1. Additional journal articles were identified from the bibliography of the studies included. At study design, all the authors agreed to exclude from final analysis data of pregnancy and perinatal complications related to: 1. prepregnancy maternal factors; 2. low technology interventions (intervention aimed to enhance fertility without any manipulation of female gametes, ie. lifestyle intervention programs, insulin sensitizing drugs, ovulation inductors, macro- and micro-supplements, intrauterine insemination, etc.); 3. specific high technology infertility treatments for male factor [including intracytoplasmic sperm injection (ICSI), specific techniques for sperm selection and/or retrieval, etc.]; 4. ovarian tissue/ovary and uterus transplantation; 5. chromosomal abnormalities and malformations of the offspring. The choice to consider a study relevant in order to be included in the current review was arbitrarily taken by each author, even if an evidence-based hierarchy was used. Exploratory studies on mechanisms of action and/or pathogenesis of any complication were included only in absence of available clinical data. Data on the efficacy of each procedure were reported only as necessary for the study aim. Any disagreement or uncertainty was resolved by discussion to reach a consensus.

**Table 1:** Key words used to study the relationship between specific procedure of ARTs for female or couple infertility and obstetric and perinatal outcomes

Intervention	Outcome
assisted reproductive technologies	antepartum hemorrhage
ART	children health
blastocyst	cesarean section
cleavage-stage	complication
controlled ovarian stimulation	delivery
controlled ovarian hyperstimulation	diabetes
embryo donation	gestational diabetes
embryo transfer	hypertension
extensive culture	hypertensive disorders
frozen-thawed	labor
gamete donation	maternal health
gestational carrier	mortality
gonadotropin	morbidity
infertility	multiple pregnancy
IVF	neonatal health
in vitro fertilization	neonatal complication
IVM	obstetric complication
in vitro maturation	offspring
oocyte donation	perinatal complication
ovarian stimulation	perinatal health
PGD	perinatal care
pre-implantation genetic diagnosis	placenta
pre-implantation genetic screening	placenta accreta
single embryo transfer	placenta previa
slow freezing	postpartum hemorrhage
sperm donation	preeclampsia
sterility	pregnancy
subfertility	pregnancy complication
surrogacy	pregnancy-induced hypertension
surrogate	prenatal care
vitrification	

The available evidence about the relationship between high technology infertility treatments and adverse obstetric and perinatal outcomes was analyzed assessing the level of evidence according to the Oxford Centre for Evidence-Based Medicine (OCEM)-Levels of Evidence 2011 guidelines [14] and the quality of evidence according to the Grading of Recommendations Assessment, Development, and Evaluation (GRADE) system [15].

## Results

**Fig. 1:**
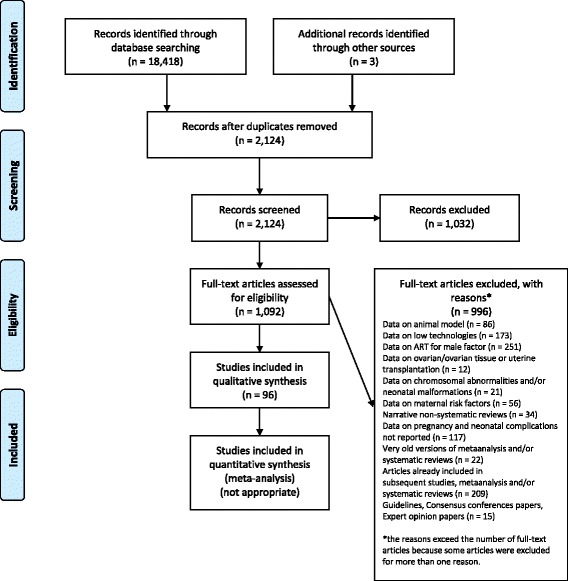
PRISMA 2009 [13] flow diagram

Figure 1 is the flow diagram of the systematic review including the numbers of studies screened, assessed for eligibility, and included in the review, with reasons for exclusions at each stage [13]. Table 2 summarizes the main risks for obstetric and perinatal adverse outcomes in women who receive ART and specific procedure for high technology infertility treatments according to the level [14] and the quality [15] of available evidence.

**Table 2:** Levels and quality of the evidences available about the relationship between specific procedure for ARTs and risk of the main obstetric and perinatal adverse outcomes

Intervention	Outcome	Evidence		Risk measures	References
		Level^a^	Quality^b^		
ART in singleton pregnancies	Compared to SCs, increased risk of:				
	Antepartum hemorrhage	3	Moderate	RR 2.11, 95 % CI 1.86 to 2.38	Qin et al., 2016 [23]
	Placental abruption	3	Moderate	RR 1.83, 95 % CI 1.49 to 2.24	Qin et al., 2016 [23]
	Postpartum hemorrhage	3	Moderate	RR 1.29, 95 % CI 1.06 to 1.57	Qin et al., 2016 [23]
	Peripartum hysterectomy	3	Moderate	aOR 5.98, 95 % CI 2.18 to 16.40	Cromi et., 2016 [21]
	GDM	3	High	RR 1.31, 95 % CI 1.13 to 1.53	Qin et al., 2016 [23]
	PIH/PE	3	Moderate	RR 1.49, 95 % CI 1.39 to 1.59	Pandey et al., 2012 [18]
	PTB	3	High	RR 1.71, 95 % CI 1.59 to 1.83	Qin et al., 2016 [23]
	Cesarean section	3	Moderate	RR 1.58, 95 % CI 1.48 to 1.70	Qin et al., 2016 [23]
	Perinatal mortality	3	High	RR 1.64, 95 % CI 1.41 to 1.90	Qin et al., 2016 [23]
	SGA	3	High	RR 1.35, 95 % CI 1.20 to 1.52	Qin et al., 2016 [23]
ART in multiple pregnancies	Compared to SCs, increased risk of:				
	Premature rupture of membranes	3	Moderate	RR 1.20, 95 % CI 1.05 to 1.37	Qin et al., 2015 [22]
	PIH	3	Moderate	RR 1.11, 95 % CI 1.04 to 1.19	Qin et al., 2015 [22]
	GDM	3	Moderate	RR 1.78, 95 % CI 1.25 to 2.55	Qin et al., 2015 [22]
	PTB	3	Moderate	RR 1.08, 95 % CI 1.03 to 1.14	Qin et al., 2015 [22]
	LBW	3	Moderate	RR 1.04, 95 % CI 1.01 to 1.07	Qin et al., 2015 [22]
	Compared to SCs, no difference in:				
	Perinatal mortality (in dichorionic twins)	3	Low	RR 1.38, 95 % CI 0.83 to 2.30	Qin et al., 2016 [23]
Number of embryos transferred	SET vs. DET, no difference in:				
	PTB	3	Low	aOR 0.83, 95 % CI 0.64 to 1.06	Pinborg et al., 2013 [9]
VTS in IVF/ICSI patients	Compared to VTS in NCs, increased risk of:				
	GDM	3	Low	aOR 3.0, 95 % CI 1.6 to 5.6	Marton et al., 2016 [35]
	IUGR	3	Low	aOR 3.0, 95 % CI 1.8 to 5.2	Marton et al., 2016 [35]
	PE	3	Low	aOR 1.6, 95 % CI 0.7 to 6.1	Marton et al., 2016 [35]
	LBW	3	Low	aOR 4.0, 95 % CI 1.8 to 7.1	Marton et al., 2016 [35]
COH	CC-IVF vs. natural-cycle IVF, increased risk of:				
	LBW	3	Low	aOR 2.09, 95 % CI 1.34 to 3.33	Nakashima et al., 2013 [43]
	Hyper^c^ vs. normal response, increased risk of:				
	LBW	3	Very Low	aOR 1.17, 95 % CI 1.05 to 1.30	Sunkara et al., 2015 [49]
	PTB	3	Very Low	aOR 1.15, 95 % CI 1.03 to 1.28	Sunkara et al., 2015 [49]
	Poor^d^ vs. normal response, no differences in:				
	LBW	3	Very Low	aOR 0.92, 95 % CI 0.79 to 1.06	Sunkara et al., 2015 [49]
	PTB	3	Very Low	aOR 0.88, 95 % CI 0.76 to 1.01	Sunkara et al., 2015 [49]
Blastocyst state of ET	Compared to cleavage-state, increased risk of:				
	PTB	3	Very Low	aOR 1.39, 95 % CI 1.29 to 1.50	Kalra et al., 2012 [61]
	VPTB	3	Very Low	aOR 1.35, 95 % CI 1.13 to 1.61	Kalra et al., 2012 [61]
	LGA	3	Low	aOR 2.22, 95 % CI 1.14 to 4.34	Mäkinen et al., 2013 [65]
Frozen-thawed ET	Compared to fresh-ET, reduced risk of:				
	LBW	3	Low	RR 0.69, 95 % CI 0.62 to 0.76	Maheshwari et al., 2012 [81]
	PTB	3	Low	RR 0.84, 95 % CI 0.78 to 0.90	Maheshwari et al., 2012 [81]
	SGA	3	Low	RR 0.45, 95 % CI 0.30 to 0.66	Maheshwari et al.,2012 [81]
	Compared to fresh-ET, increased risk of:				
	Placenta accreta	3	Low	aOR 3.16, 95 % CI 1.71 to 6.23	Ishihara et al., 2014 [89]
	PIH/PE	3	Low	aOR 1.58, 95 % CI 1.35 to 1.86	Ishihara et al., 2014 [89]
	LGA	3	Moderate	aOR 1.54, 95 % CI 1.31 to 1.81	Pinborg et al., 2014 [98]
	Macrosomia	3	Moderate	aOR 1.64, 95 % CI 1.26 to 2.12	Pinborg et al., 2014 [98]
Vitrified oocytes	Compared to fresh oocytes, no differences in:				
	GDM	3	Very low	aOR 0.86, 95 % CI 0.56 to 1.31	Cobo et al., 2014 [107]
	PIH	3	Very low	aOR 0.84, 95 % CI 0.59 to 1.20	Cobo et al., 2014 [107]
	LBW	3	Very low	aOR 1.06, 95 % CI 0.78 to 1.42	Cobo et al., 2014 [107]
	PTB	3	Very low	aOR 0.70, 95 % CI 0.50 to 1.00	Cobo et al., 2014 [107]
Vitrified embryos (blastocysts)	Compared to fresh-blastocysts, reduced risk of:				
	LBW	3	Very low	aRR 0.67, 95 % CI 0.58 to 0.78	Li et al., 2014 [115]
	PTB	3	Very low	aRR 0.86, 95 % CI 0.77 to 0.96	Li et al., 2014 [117]
	SGA	3	Very low	aRR 0.60, 95 % CI 0.53 to 0.68	Li et al., 2014 [115]
IVM of oocytes	Compared to in vivo matured oocytes (vs. IVF and vs. ICSI), no differences in:				
	LBW	3	Very low	3 % vs. 10 % vs. 14 % (*p*: NS)	Buckett et al., 2007 [123]
	PTB	3	Very low	38 % vs. 30 % vs. 36 % (*p*:NA)	Buckett et al., 2007 [123]
PGD	Blastocyst biopsy and frozen ET vs. cleavage-stage biopsy and fresh ET, increased risk of:				
	PIH	4	Very low	aOR 4.85, 95 % CI 1.34 to 17.56	Jing et al., 2016 [136]
Oocyte donation	Compared to autologous oocyte in IVF singletons, increased risk of:				
	PIH	3	Moderate	aOR 2.30, 95 % CI 1.60 to 3.32	Storgaard et al., 2016 [155]
	PE	3	Moderate	aOR 2.11, 95 % CI 1.42 to 3.15	Storgaard et al., 2016 [155]
	PTB	3	Moderate	aOR 1.75, 95 % CI 1.39 to 2.20	Storgaard et al., 2016 [155]
	LBW	3	Moderate	aOR 1.53, 95 % CI 1.16 to 2.01	Storgaard et al., 2016 [155]
	Postpartum hemorrhage	3	Low	aOR 2.40, 95 % CI 1.49 to 3.88	Storgaard et al., 2016 [155]
Sperm donation	Singletons with donor semen vs.singletons with partner semen, increased risk of:				
	PE	3	Very low	18.2 % vs. 0 % (*p* < 0.05)	Salha et al., 1999 [159]
Embryo donation	NA				
Gestational carrier	Surrogate singletons vs. control IVF-singleton pregnancies, no differences in:				
	PTB	4	Low	0–11.5 % vs. 14 % (*p*:NA)	Söderström-Anttila et al., 2016 [162]
	LBW	3	Low	0–11.1 % vs. 13.6–14.0 % (*p*:NA)	Söderström-Anttila et al., 2016 [162]

*aRR* adjusted relative risk, *aOR* adjusted odds ratio, *ART* assisted reproductive technologies, *CC* clomiphene citrate, *COH* controlled ovarian hyperstimulation, *DET* double embryo transfer, *EPTB* early preterm birth, *ET* embryo transfer, *GDM* gestational diabetes mellitus, *IUI* intrauterine insemination, *IVM* in vitro maturation, *LBW* low birth weight, *LGA* large for gestational age, *LTIFE* low technology interventions for fertility enhancement, *NA* not available data, *NC* natural conception, *NP* not performed, *NS* not significant, *PE* preeclampsia, *PGD* pre-implantation genetic diagnosis, *PIH* pregnancy-induced hypertension, *PTB* preterm birth, *RR* relative risk, *SET* single embryo transfer, *SGA* small for gestational age, *VLBW* very low birth weight, *VTS* vanishing twin syndrome

^a^assessed following the Oxford Centre for Evidence-Based Medicine (OCEM) - Levels of Evidence 2011 guidelines [14]

^b^assessed using the Grading of Recommendations Assessment, Development, and Evaluation (GRADE) system [15]

^c^more than 20 oocytes

^d^less or equal to 3 oocytes

### Overall ART-related complications

#### Singleton pregnancies

Many data from systematic reviews with and without meta-analyses have demonstrated that ART singletons are at increased risk of pregnancy and perinatal complications. In 2004, an initial systematic review of controlled studies found a relative risk of 3.27 (95 % CI 2.03 to 5.28) for early-preterm birth (EPTB) (< 32 weeks) in singleton pregnancies from assisted conceptions [16]. Singleton pregnancies resulting from in vitro fertilization (IVF) presented higher rates of worse obstetric outcome, compared with NC singletons of couples matched for maternal age, showing an increase in perinatal mortality [odd ratio (OR) 2.40, 95 % confidence interval (CI) 1.59 to 3.63], PTB at < 33 weeks’ gestation (OR 2.99, 95 % CI 1.54 to 5.80), PTB at < 37 weeks’ gestation (OR 1.93, 95 % CI 1.36 to 2.74), very-low preterm birth (VLBW) (<1500 g) (OR 3.78, 95 % CI 4.29 to 5.75), and SGA (OR 1.59, 95 % CI 1.20 to 2.11) [17]. It is noteworthy that this review included in the final analysis studies in which the control arm was composed of NCs and after ovulation induction strategies. In 2012, an extensive systematic review with meta-analysis of 20 matched and 10 unmatched cohort studies, most having high quality and adjusted for important confounders, concluded that singleton IVF/intracytoplasmic sperm injection (ICSI) pregnancies are associated with higher risks of antepartum hemorrhage [relative risk (RR) 2.49, 95 % CI 2.30 to 2.69], pregnancy-induced hypertension (PIH)/preeclampsia (PE) (RR 1.49, 95 % CI 1.39 to 1.59), gestational diabetes mellitus (GDM) (RR 1.48, 95 % CI 1.33 to 1.66), caesarean section (RR 1.56, 95 % CI 1.51 to 1.60), PTB (RR 1.54, 95 % CI 1.47 to 1.62), LBW (RR 1.65, 95 % CI 1.56 to 1.75), SGA (RR 1.39, 95 % CI 1.27 to 1.53), admission to neonatal intensive care unit (NICU) (RR 1.58, 95 % CI 1.42 to 1.77), and perinatal mortality (RR 1.87, 95 % CI 1.48 to 2.37) than NC [18]. Also in this last meta-analysis, only in some of the included studies were the pregnancies resulting from ovulation induction excluded from non-IVF/ICSI conceptions [18].

The most recently published meta-analysis about the risk of pregnancy-related complications and adverse perinatal outcomes in singleton pregnancies obtained with ART involves 50 cohort studies for a total of 161,370 ART singleton compared with 2,280,241 NC singleton pregnancies [19]. This meta-analysis revealed that the singleton ART pregnancies experienced a significantly increased risk of placenta previa (RR 3.71, 95 % CI 2.67 to 5.16), placental abruption (RR 1.83, 95 % CI 1.49 to 2.24), antepartum hemorrhage (RR 2.11, 95 % CI 1.86 to 2.38), and postpartum hemorrhage (RR 1.29, 95 % CI 1.06 to 1.57). The increased risk for PIH, GDM, caesarean sections, PTB, LBW, SGA and perinatal mortality was confirmed and resulted not different from previous meta-analytic data [19]. Of note, the risk of EPTB (RR 2.12, 95 % CI 1.73 to 2.59) and of VLBW (RR 2.12, 95 % CI 1.84 to 2.43) was two-fold higher in ART conceptions than in NC [19]. The results of this study are consistent with those of the previous reviews but it presents important strengths, such as the large sample size, 64 % of the included studies were considered of high methodological quality and the association between ART and obstetric risk persisted and remained statistically significant in sensitivity analysis based on various exclusion criteria [19]. Nevertheless, as relevant biases, patients who achieved a pregnancy with ovulation induction and/or intrauterine insemination (IUI) were included in the NC category, resulting in an underestimation of the association between ART and adverse outcomes. When data were restricted to studies that did not include these patients in the NC group, the risk of GDM, placental abruption, PTB, EPTB, LBW, VLBW, and perinatal mortality resulted in a further increase. Unfortunately, more than half of the included studies did not specify whether they were included, thereby restricting this subgroup analysis [19]. Finally, recent retrospective studies confirmed in ART pregnancies a risk of PIH/PE about 20 % higher (aOR 1.17, 95 % CI 1.10 to 1.24) [20] and demonstrated a risk of peripartum hysterectomy about six-fold increased (OR 5.98 95 % CI 2.18 to 16.40) in comparison with non-ART pregnancies [21].

#### Multiple pregnancies

Initial systematic reviews with meta-analyses [16, 17] demonstrated that ART twins had a higher risk of PTB compared with NC twins but the perinatal mortality, contrary to singletons, was unchanged [17] or reduced [16].

A recent meta-analysis including 39 cohort studies explored the risk of adverse pregnancy outcomes in ART conceptions compared with NC in a sample of 146,008 multiple births [22]. ART were associated with a higher risk of premature rupture of membranes (RR 1.20, 95 % CI 1.05 to 1.37), PIH (RR 1.11, 95 % CI 1.04 to 1.19), GDM (RR 1.78, 95 % CI 1.25 to 2.55), PTB (RR 1.08, 95 % CI 1.03 to 1.14), EPTB (RR 1.18, 95 % CI 1.04 to 1.34), LBW (RR 1.04, 95 % CI 1.01 to 1.07), and VLBW (RR 1.13, 95 % CI 1.01 to 1.25) when compared to NC [22]. When data were restricted to matched and/or high quality studies, the risk of PIH, GDM, PTB, and LBW/VLBW increased further. Moreover, the results of main outcomes had a significant heterogeneity among studies and were significantly influenced by inclusion/exclusion of the pregnancies achieved after ovulation induction with and without IUI in NCs.

In all meta-analytic data [16, 17, 22, 23] perinatal mortality was not increased. A recent cohort study [24], however, reported an adjusted risk of perinatal death for twins 45 and 85 % lower among ART births than fertile or subfertile births, respectively. This finding can be explained because of the decreased proportion of monochorionic twins in ART pregnancies (2–7 % vs. 22–30 %) [25] showing that in twins studies it could be crucial to control/adjust data for zygosity. Furthermore, ART monochorionic twins are also at increased risk of adverse perinatal outcomes compared with spontaneous monochorionic twins. In fact, in monochorionicity ART appears to increase the already high risk of PTB <32 weeks (OR 2.9, 95 % CI 1.1 to 7.3) and VLBW (OR 5.9, 95 % CI 2.5 to 1.49) [26].

One way to overcome the zygosity as confounder could be to perform comparisons restricted to different-sex twin pairs in order to exclude all the monochorionic twins. To this regard, a recent meta-analysis [23] including 15 cohort studies and involving 6,420 dichorionic twins after ART and 13,650 dichorionic NC twins confirmed a risk of placenta previa (RR 2.99, 95 % CI 1.51 to 5.92), PTB (RR 1.13, 95 % CI 1.00 to 1.29), VPTB (RR 1.39, 95 % CI 1.07 to 1.82), LBW (RR 1.11, 95 % CI 1.00 to 1.23), and elective cesarean sections (RR 1.79, 95 % CI 1.49 to 2.16) significantly increased in ART pregnancies. Very recently, a nationwide registry based study demonstrated in women who conceived dizygotic twins after IVF/ICSI rates of PIH lower than after NC (aOR 0.74, 95 % CI 0.66 to 0.83) [27]. This did not translate into a difference in PE or delivery mode, and there was no evidence of a difference in PTB rates because women delivered prematurely in 51 % of cases both after IVF/ICSI and after NC [27].

### Number of embryos transferred

An excellent meta-analysis of individual data from RCTs demonstrated that the elective single embryo transfer (SET) is less effective (of about 50 %) than double embryo transfer (DET) in women aged more than 33 years but not in younger, and that the odds of a multiple live birth in women randomized to eSET were significantly smaller than for women who received DET [28]. After eSET, the adjusted risk to deliver a LBW baby (aOR 0.36, 95 % CI 0.15 to 0.87) and to have a PTB (aOR 0.33, 95 % CI 0.20 to 0.55) was significantly reduced, whereas the adjusted odds of a term singleton birth after eSET were almost five times higher than those after DET (aOR 4.93, 95 % CI 2.98 to 8.18) [28]. Data obtained from the US National Assisted Reproductive Technology Surveillance System, a retrospective population-based study analyzed the 82,508 fresh autologous ART cycles, confirmed that a higher chance of a good perinatal outcome, defined as a live birth at or after 37 weeks of gestation of a normal birth weight (2,500 g or greater) singleton, is associated with SET in patients with a favorable prognosis who were aged younger than 35 years [29]. The same conclusions were obtained after the analysis of the 2013 data, i.e. increasing use of elective SET, when clinically appropriate (typically age < 35 years), might reduce multiple births and related adverse consequences of ART [7]. Moreover, the average rate of SET was 21.4 % also among good prognosis patients and varied considerably among States (from 4 to 77.5 %) [7].

Irrespective of the risk related to the multiple pregnancies, the number of embryos transferred can be an independent and potential factor influencing the obstetric and perinatal outcomes. Available data were very heterogeneous showing better perinatal outcomes [30, 31] or no difference [32, 33] between SET singletons and DET singletons. A meta-analysis demonstrated no difference in the incidence of PTB (aOR 0.83, 95 % CI 0.64 to 1.06) between singletons born after SET and singletons born after DET [9]. However, an effect of vanishing twin syndrome (VTS) [34], as consequence of DET or multiple embryo transfer, cannot be excluded. This is particularly true in consideration of the higher risk of pregnancy complications observed in VTS pregnancies from IVF/ICSI cycles in comparison with VTS pregnancies from NC [35]. A large cohort study including 7,757 deliveries following IVF/ICSI procedures found more adverse outcomes among VTS survivors occurring after 8 weeks of gestation, even after adjustment for maternal age and parity [36]. However, that study did not differentiate between “true” (first trimester) cases of the VTS and those occurring at the second trimester since embryonic losses from 9 weeks until midgestation were grouped together, and a case-mix of di-and monochorionic twins is probable. On the other hand, similar maternal and perinatal complications, such as gestational duration and birth weights, were observed between VTS survivors and singletons in a retrospective series including IVF/ICSI patients when only well selected patients with initial heart beat on both embryos and with clear chorionicity were included [37].

In singletons with a ‘vanishing co-twin’, the risk of PTB (aOR 1.73, 95 % CI 1.54 to 1.94) and of other adverse perinatal outcomes (aOR ranging from 1.73 to 2.88) was significantly higher than in singletons from a single gestation [38]. The obstetric and perinatal risks in VTS seem to increase in accordance with the number of reduced fetuses [38]. Other case–control and cohort studies on singleton deliveries after IVF/ICSI showed that VTS was associated with higher risk of vaginal bleeding and preterm premature rupture of membranes, and that VTS survivors had a birth weight significantly lower, and a rate of LBW and SGA about double when compared to singleton controls [39–41]. Of note, no difference was observed in terms of duration of gestation suggesting a direct effect of the VTS on the survived embryos/fetus [40].

A well designed retrospective study on a very large cohort of 252,994 singleton deliveries evaluated the pregnancy and perinatal outcomes, adjusted for confounders such as fertility treatment and maternal age, in VTS in comparison with those recorded in singletons and dichorionic twins [42]. VTS was found to be an independent risk factor for several adverse perinatal outcomes [42]. Specifically, the risk of GDM (aOR 1.4, 95 % CI 1.01 to 2.0), intrauterine growth restriction (IUGR) (aOR 2.7, 95 % CI 1.7 to 4.3), VLBW (aOR 6.9, 95 % CI 4.7 to 10.2), low Apgar scores (aOR 1.9, 95 % CI 1.1 to 3.3), and perinatal mortality (aOR 2.4, 95 % CI 1.2 to 4.6) was significantly increased in VTS when compared with singletons. In addition, VTS significantly influenced also the risk of malpresentation, placental abruption, and cesarean section [42]. However, that study [42] included mainly NC, and the effect of VTS in IVF/ICSI vs. NC pregnancies was not tested. To this regard, a recent retrospective matched (for maternal age, previous gravidity, parity, and prepregnancy BMI) analysis demonstrated that the incidence of the VTS in infertile IVF/ICSI patients is lower than in NC (12.6 % vs. 18.2 %, respectively; estimated for twin pregnancies) but the perinatal outcomes resulted worse. In fact, the risk of GDM (aOR 3.0, 95 % CI 1.6 to 5.6 vs. aOR 0.46, 95 % CI 0.2 to 1.1) and of PE (aOR 1.6, 95 % CI 0.7 to 6.1 vs. aOR 1.00, 95 % CI 0.8 to 1.8) in VTS pregnancies was very high in IVF/ICSI patients but lower or unchanged in NC women [35]. Also the incidence of IUGR (aOR 3.0, 95 % CI 1.8 to 5.2 vs. aOR 9.2, 95 % CI 5 to 22) and of LBW (aOR 4.0, 95 % CI 1.8 to 7.1 vs. aOR 2.1, 95 % CI 1.6 to 4.0) in VTS pregnancies was higher in IVF/ICSI patients than in NC women [35]. Very interesting data from multiple logistic regression analyses that identified in VTS pregnancies obtained after IVF/ICSI cycles a risk of IUGR 28-fold higher (aOR 28.2, 95 % CI 2.2 to 14.5) [35].

### Controlled ovarian hyperstimulation (COH)

In comparison with children born after natural-cycle IVF, IVF children conceived after stimulation with clomiphene citrate (CC) and with CC plus gonadotropins had a higher risk of LBW [43]. On the other hand, IVF children conceived after ovarian stimulation with gonadotropins alone did not have a higher risk of LBW compared to natural-cycle [43] confirming IUI data [44]. In addition, no effect of the duration of stimulation, amount of drug administered, and number of oocytes retrieved was detected on SGA or LBW in a cohort study on 32,000 singletons born from gonadotropin IVF/ICSI cycles [45]. A retrospective cohort study on 392 women less than 40 years of age demonstrated the safety, in terms of obstetric and neonatal complications, of the use of gonadotropin-releasing hormone (GnRH) agonist to trigger ovulation in antagonist cycles. Specifically, there were no significant differences in the rate of maternal (27.6 vs. 20.8 %) or neonatal complications (19.7 vs. 20.0 %) between the GnRH agonist and hCG [46]. In addition, luteal GnRH antagonist administration in women with severe early ovarian hyperstimulation syndrome (OHSS) is associated not only with not different live birth rates but also with similar duration of gestation (36.9 ± 0.9 vs. 36.9 ± 2.4 weeks) and neonatal weight (2392 ± 427 vs. 2646 ± 655 g) when compared to patients who do not receive the treatment [47]. However, in patients undergoing COH using the GnRH antagonist protocol, GnRH agonist trigger were found to be independent risk factors for ectopic pregnancy [48].

The ovarian response to COH has been also related to pregnancy complications. A recent observational United Kingdom (UK) study [49] on 402,185 IVF cycles and 65,868 singleton live birth outcomes detected a risk of PTB (aOR 1.15, 95 % CI 1.03 to 1.28), EPTB (aOR 1.30, 95 % CI 1.03 to 1.64), LBW (aOR 1.17, 95 % CI 1.05 to 1.30), and VLBW (aOR 1.23, 95 % CI 0.97 to 1.55) higher in women with a high number (>20) of oocytes retrieved when compared with women with a normal response (10–15 oocytes) [49]. Moreover, the observation that higher rates of LBW infants in singleton gestations after IVF cycles was only present in patients who had a fresh ET when compared with those with frozen-thawed cycles [50] has suggested that the gonadotropin stimulated multifollicular development and the production of supraphysiologic levels of sex steroid hormones immediately before embryo implantation might represent an independent contributing factor to that increased risk (and not only an effect of fresh or frozen embryos as below detailed). In fact, in IVF cycles the elevated peak serum estradiol (E_2_) levels on the day of the human chorionic gonadotropin (hCG) trigger were closely related to the risks of SGA and PE in singleton pregnancies [51, 52]. Thus, the supraphysiologic hormonal milieu at the time of implantation and placentation during a fresh IVF cycle may modulate trophoblast invasion and lead to abnormal placentation, whereas hormonal levels in a frozen ET cycle are much more akin to the endocrine environment of NC. This hypothesis has been tested by a small cohort study on a population at high risk for OHSS for high E_2_ levels; the elective cryopreservation of all embryos with subsequent thawed ET reduced significantly the risk of PE (21.9 % vs. 0 %, respectively; OR not calculable), and to deliver SGA infants (OR 0.09, 95 % CI 0.01 to 0.65) as compared with the patients who had fresh ET [53]. However, the published case–control studies on the obstetric and perinatal outcomes in patients with OHSS showed divergent results [54–56]. In line with previous data, no increased risk of the adverse outcomes among women with a poor ovarian response (≤ 3 oocytes) compared with women with a normal response was observed [49], even if several important confounders, such as smoking, body mass index (BMI), polycystic ovarian syndrome (PCOS), and gonadotropin doses were not available for an optimal data adjustment. On the other hand, a retrospective cohort study of the Society for Assisted Reproductive Technology (SART) on 14,086 patients demonstrated an inverse relationship between maximal basal follicle stimulating hormone (FSH) levels and the risk for PTB and LBW in singleton IVF gestations with the lowest risk of PTB (aRR 0.87, 95 % CI 0.76 to 1.01) and of LBW (aRR 0.89, 95 % CI 0.73 to 1.04) in patients with the lowest ovarian reserve, defined as highest serum basal and/or CC-induced FSH levels [57].

Finally, the hitherto published literature regarding the relation between OHSS and pregnancy complications is limited and controversial [56]. This disparity in the observed outcomes probably results from the inclusion of patients with OHSS, not exclusively limited to those with significant increase in vascular permeability, as reflected by third space fluid accumulation necessitating drainage. A recent study [58] has demonstrated that severe OHSS, complicated by third space fluid sequestration necessitating drainage, is not associated with adverse late pregnancy outcome (i.e. IUGR and PIH/PE), except for PTB. Therefore, following resolution of the OHSS, pregnancies should be regarded as any pregnancy resulting from IVF treatment, with special attention to identify and treat PTB.

### Blastocyst vs. cleavage state ET

In a first systematic review with meta-analysis [9], the risk of PTB was not significantly different in singletons conceived after day 5 vs. day 2 embryo culture (aOR 1.14, 95 % CI 0.80 to 1.64). A further meta-analysis [59] aimed to assess specifically the risk of pregnancy complications in singleton pregnancies resulting from ET at the blastocyst stage (days 4–5 or 5–6) compared with those resulting from ET at the cleavage stage (days 2–3 or day 3 or days 2–4) demonstrated, after data synthesis of 7 retrospective cohort studies, that pregnancies occurring as a result of ET at the blastocyst stage were associated with a higher risk of PTB (RR 1.27, 95 % CI 1.22 to 1.31) and VPTB (RR 1.22, 95 % CI 1.10 to 1.35) delivery and a lower risk of SGA [59]. However, these data were not adjusted for confounders and included very different study protocols. Another more recent meta-analytic study [60] aimed to evaluate the perinatal outcomes among singleton births following blastocyst stage (day 5 or 6) ET compared with cleavage stage (day 2–4) ET and included 6 observational studies with low to moderate risk of bias. It demonstrated, after adjusting data for main confounders, that only the risk of PTB (aOR 1.32, 95 % CI 1.19 to 1.46) was significantly higher among births after blastocyst transfer than after cleavage stage transfer [60]. No further difference in perinatal/neonatal outcome was observed [60].

At the moment, the most complete data on maternal and child health adjusted for confounders were from a large retrospective cohort study [61] comparing 46,288 neonates born after cleavage-stage ET to 22,751 neonates born after blastocyst stage ET, and showing an increased risk for PTB (aOR 1.39, 95 % CI 1.29 to 1.50) and VPTB (aOR 1.35, 95 % CI 1.13 to 1.61) in singletons neonates born after transfer of embryos at blastocyst stage vs. cleavage stage [61]. These results were confirmed also in twins (aOR 1.81, 95 % CI 1.63 to 2.00; and aOR 1.75, 95 % CI 1.50 to 2.04 for PTB and VPTB, respectively) [61]. However, pregnancies that did not result in a live birth were not analyzed and the number of embryos transferred was not considered. To this regard, a retrospective cohort study analyzed the transfer of 693 fresh single cleavage embryos comparing to 850 fresh single blastocysts [62]. A higher live birth rate (33.5 % vs. 13.8 %) was detected after single blastocysts transfer and after adjusting data for several confounders, while no effect of the extended culture was observed on obstetric or perinatal outcomes [62]. These data have been confirmed also more recently in a retrospective matched case–control study by the same researchers [63] and by a large retrospective population-based study including a total of 50,788 infants (43,952 singleton and 3,418 twin deliveries) [64]. Conversely, there were lower odds of PTB among twins (aOR 0.80, 95 % CI 0.70 to 0.93) conceived after blastocyst transfer than after cleavage stage ET [64].

An important issue observed in the studies comparing the transfer of embryos at blastocyst vs. cleavage state was the discrepancy between the increased incidence in PTB and the similar incidence of LBW neonates in singletons [61]. Regarding this, an effect of the extended culture on the neonatal weight has been demonstrated. In fact, the incidence of SGA in neonates born after extended embryo culture compared with cleavage stage ET was significantly lower (OR 0.80, 95 % CI 0.74 to 0.87) [61]. The effect of extended embryo culture on fetal growth has been specifically assessed in a cohort study on 1,079 singleton neonates born after fresh ET [65]. The risk of large-for-gestational age (LGA) (but not for SGA) adjusted for mother’s age, BMI, parity, type of treatment (IVF or ICSI), main cause of infertility and embryo culture period was increased after extended embryo culture (aOR 2.22, 95 % CI 1.14 to 4.34), and the length of the embryo culture was a strong and significant factor determining the gestation and gender-adjusted birth weight of the IVF babies [65].

On the other hand, a recent retrospective registry study [66] (including 4,819 singletons born after blastocyst ET, 25,747 after cleavage stage ET, and 1,196,394 after NC) demonstrated, after adjusting data for several confounders, a risk of perinatal mortality (aOR 1.61, 95 % CI 1.14 to 2.29) higher in singletons born after blastocyst ET vs. cleavage stage ET, although a lower incidence of LBW (aOR 0.83, 95 % CI 0.71 to 0.97) and SGA (aOR 0.71, 95 % CI 0.56 to 0.88) children was confirmed. The risk of placenta previa (aOR 2.08, 95 % CI 1.70 to 2.55) and placental abruption (aOR 1.62, 95 % CI 1.15 to 2.29) was higher in pregnancies after blastocyst ET vs. pregnancies after cleavage stage ET [66].

The findings of obstetric and perinatal complications in pregnancies achieved after ET at the blastocyst stage are unexpected because good quality embryos from women with the best prognosis tend to be selected for extended culture. The specific mechanism underlying the association between extended embryo culture and PTB is unknown. It could be the result of the transfer at blastocyst stage inducing a defective implantation for asynchrony between the endometrium and embryo [67], trigger genetic and epigenetic modifications in the pre-implantation embryos due to the culture medium of the extended culture [67, 68] and/or in decidual/trophodermal cells due to abnormal embryo-endometrium interaction [69]. In fact, extended culture up to blastocyst stage increases the incidence of monozygotic twins (OR 3.04, 95 % CI 1.54 to 6.01) and the male-to-female ratio (OR 1.29, 95 % CI 1.10 to 1.51 [70], and both are associated with PTB [71]. Although an effect of the procedure of embryo freezing and thawing cannot be excluded (see below), all these factors, alone and/or in concert, can be responsible for the higher risk of PTB detected in children born after blastocyst transfer [72].

Well-designed studies [73–75] have recently evaluated the effect of different types of in vitro culture medium and of the duration of in vitro culture on obstetric and perinatal outcomes in IVF/ICSI populations showing conflicting results, suggesting that data cannot be generalized for all commercial culture media. The different culture medium (Medicult vs. Vitrolife) and the duration of culture (day 3 vs. day 5) had no effect on singleton newborns birthweight, as well as on the incidence of PTB and VPTB, was detected on a large population of 2,098 infertile women treated with fresh IVF and ICSI cycles after controlling for confounders [75]. Unfortunately, all these studies had a retrospective design and the risk of residual confounding cannot be excluded [72].

### Frozen-thawed vs. fresh ET

The proportion of frozen ET has increased in consideration of the better reproductive outcomes compared to IVF fresh cycles [76, 77]. Analyzing the SART database from 2008 to 2011, odds of extra-uterine pregnancy 65 % lower in women who had a frozen compared with a fresh transfer in autologous cycles were observed [78], that risk is lower for day 5 blastocyst vs. day 3 embryos [79].

The first systematic reviews [80] available in the literature, including mostly pregnancy studies achieved by using slow-frozen embryos and only a few vitrification studies, concluded that the risk of adverse obstetric outcome was similar between cryopreserved embryos/oocytes and NC children or fresh ET. A more recent systematic review with meta-analysis [81], including 11 observational studies, suggested that singleton pregnancies arising from frozen embryos seem to offer better obstetric and perinatal outcome than those obtained after fresh oocyte cycles. The clinical benefits regarded the risk of antepartum hemorrhage (RR 0.67, 95 % CI 0.55 to 0.81), PTB (RR 0.84, 95 % CI 0.78 to 0.90), SGA (RR 0.45, 95 % CI 0.30 to 0.66), LBW (RR 0.69, 95 % CI 0.62 to 0.76) and perinatal mortality (RR 0.68, 95 % CI 0.48 to 0.96) [81]. The use of frozen ET was also subsequently confirmed to be a predictor of reduced risk of PTB when compared to fresh ET after adjusting data for main confounders (aOR 0.85, 95 % CI 0.76 to 0.94) [9]. However, a significant heterogeneity in the technique of freezing (slow freezing vs. vitrification), in embryo stage (cleavage stage vs. blastocyst stage vs. both), and in regimen in replacement cycles (natural cycles vs. hormone replacement cycles) was observed among studies. In addition, a bias due to difference in prognosis between populations cannot be excluded. In fact, although the risk of pregnancy complications was higher in the paired comparison of the same patient who conceived after fresh vs. subsequent frozen-thawed ET (including donor cycles) [82], it is likely that the frozen embryo populations are more likely composed of the better prognosis patients. Similarly, also the selection bias due to the physical effects of freezing and thawing on the worse embryos cannot be excluded.

In a large retrospective cohort study [83], the perinatal outcome of 6,647 singletons conceived after frozen-thawed ET were compared with 42,242 singletons born after fresh ET and 288,542 singletons born after spontaneous conception. Data, adjusted for several confounding factors, confirmed that singletons conceived after frozen-thawed ET had better perinatal outcomes than after fresh ET [83]. Specifically, albeit the perinatal outcomes were worse when compared to NC, singletons born after frozen-thawed ET had a lower risk of LBW (aOR 0.81, 95 % CI 0.71 to 0.91), PTB (aOR 0.84, 95 % CI 0.76 to 0.92) and SGA (aOR 0.72, 95 % CI 0.62 to 0.83) when compared with singletons born after fresh ET. However, after frozen-thawed ET, the perinatal mortality resulted increased when compared with both fresh ET (aOR 1.49, 95 % CI 1.07 to 2.07) and NC (aOR 1.39, 95 % CI 1.03 to 1.87) [83]. The increased perinatal mortality rate has been showed only in few studies [84] but not in others [85–88]. Specifically, the perinatal mortality rate resulted 25.2 per 1,000 births for fresh IVF/ICSI vs. 17.5 per 1,000 for frozen-thawed cycles [88].

A Japanese large-scale registry-based study not only confirmed that SET of frozen-thawed blastocyst was associated with significantly lower odds of PTB, LBW and SGA, but, for the first time, suggested a risk three-fold higher of placenta accreta (aOR 3.16, 95 % CI 1.71 to 6.23) after frozen-thawed ET than after fresh ET [89]. A study [90] explored specifically this complication in patients using IVF/ICSI, with autologous or donor oocytes, undergoing fresh or cryopreserved transfer. After multivariate analysis (including many predictors such as non-Caucasian race, uterine factor infertility, prior myomectomy and placenta previa), a significant and specific association between frozen-thawed ET and placenta accreta (aOR 3.20, 95 % CI 1.14 to 9.02) was detected [90]. On the other hand, a decreased risk of placenta previa (RR 0.71, 95 % CI 0.53 to 0.95) and placental abruption (RR 0.44, 95 % CI 0.24 to 0.83) in pregnancies after frozen thawed ET was reported [81]. Unfortunately, the demographics of patients having these complications is lacking in these studies.

The frozen-thawed embryo cycles showed an increased association with PIH/PE (aOR 1.58, 95 % CI 1.35 to1.86) [89], similarly to data from the Swedish IVF registry [84]. In comparison with pregnancies following fresh cycles, those following frozen-thawed ET had a higher risk of PIH/PE with a risk difference of 1.8 % (95 % CI 1.2 to 2.8) and 5.1 % (95 % CI 3.0 to 7.1) in singleton and twin pregnancies, respectively [91]. These data were confirmed when compared with fresh cycle pregnancies by the same mother after sibling analysis (OR 2.63, 95 % CI 1.73 to 3.99) [91] and, interestingly, also in PCOS patients [92]. In fact, a large multicenter RCT on a total of 1,508 infertile women with PCOS assigned to undergo either fresh-embryo transfer or embryo cryopreservation (vitrification) followed by frozen-embryo transfer demonstrated an incidence of PE significantly higher with frozen-embryo transfer [rate ratio (ReRa) 3.12, 95 % CI 1.26 to 7.73] [92].

The risk of post-term birth (aOR 1.40, 95 % CI 1.27 to 1.55), LGA (aOR 1.45, 95 % CI 1.27 to 1.64), and macrosomia (aOR 1.58, 95 % CI 1.39 to 1.80) also increased [83, 89]. Of note, even if not statistically significant, the risk of LGA was higher in boys than in girls (41 % vs. 17 %) [83] confirming previous data that showed an odd for LBW in frozen-thawed ET significantly higher in female than that in male neonates [93]. The first paper reporting the increased risk of LGA newborns in frozen-thawed ET compared with fresh ET, irrespective of maternal BMI and abnormal oral glucose tolerance test (OGTT), was published in 2010 [85]. At the moment, there is not a conclusive hypothesis for explaining that finding and many potential explanations of the increased rate of LGA in frozen-thawed embryos have been proposed. Different media used for culturing of human embryos can differently affect the birth-weight of the newborns [94] with an effect of the embryo freezing and thawing [95]. However, the effect on human embryos of the many components of the culture media is not known. A clinical study [96] demonstrated no difference in birth-weight between two culture media but a higher birth-weight after embryo freezing and thawing in both groups when compared with fresh cycles suggesting generic effect of cryoprotectants on enzymatic pattern involved in epigenetic programming [97]. However, a specific effect of media for extended culture cannot be excluded since babies born after day 5–6 transfer had significantly higher risk of LGA compared with babies born after day 2 transfer [65] (see before).

Recently, a national register-based controlled cohort study with further meta-analysis was designed in order to clarify whether the “large baby syndrome” was caused by intrinsic maternal factors or related to the freezing/thawing procedures [98]. In order to avoid biases due to different frozen techniques and embryo culture/stage, only singletons born after slow freezing and ET of cleavage stage embryos (day 2 or 3) were included in the meta-analysis [98]. The increased risk of LGA in frozen-thawed embryos singletons was firstly confirmed also in a sibling cohort after adjusting data for maternal age, parity and year of birth (aOR 3.45, 95 % CI 1.33 to 8.33) [98]. The pooled risk for LGA (aOR 1.54, 95 % CI 1.31 to 1.81) and macrosomia (aOR 1.64, 95 % CI 1.26 to 2.12) was confirmed in frozen-thawed embryos vs. fresh embryos [98]. These risks were the same extension as when compared to NC [98]. Unfortunately, these data were not adjusted for maternal BMI and GDM that can act as residual confounders [98].

A recent retrospective UK cohort study [99] analyzed a total of 112,432 cycles (95,911 fresh and 16,521 frozen cycles) using multivariate logistic regression and detected no association between type of embryo transferred (frozen vs. fresh) and PTB (aRR 0.96, 99.5 % CI 0.88 to 1.03) and VPTB [aRR 0.86, 99.5 % CI 0.70 to 1.05). On the other hand, the odds of LBW (aRR 0.73, 99.5 % CI 0.66 to 0.80) and VLBW (aRR 0.78, 99.5 % CI 0.63 to 0.96) were lower after frozen ET, and those of having a LGA was higher (aRR 1.64, 99.5 % CI 1.53 to 1.76) [99]. Moreover, that data were anonymized and regarded outcomes of IVF cycles and not of IVF patients, and did not included pregnancy complications. Thus, a bias due to more cycles of the same patient cannot be excluded, and the influence of the obstetric complications on perinatal outcomes cannot be assessed.

Finally, two retrospective recent cohort studies suggest that frozen cycles are associated with a reduction of the incidence of ectopic pregnancies and monozygotic twins [100, 101]. Specifically, a registry study [100] based on a total of 44,102 pregnancies from Australian and New Zealand Assisted Reproduction Technology Database demonstrated that the SET of frozen blastocyst is associated with lowest risk of ectopic pregnancy (aOR 0.70, 95 % CI 0.54 to 0.91; vs. fresh blastocyst transfer), and that the characteristics influencing that risk were the number of embryo transferred, and the use of fresh and/or cleavage stage embryos. In addition, the analysis of 6,103 cycles revealed that frozen ET was associated with a significant reduction in monozygotic twins incidence (aOR 0.48, 95 % CI 0.29 to 0.80) [101].

### Vitrified vs. slow frozen vs. fresh cycles

Initially, there were some safety concerns about old vitrification procedures because the use of very high concentrations of cryoprotectants required to optimize the efficacy could induce toxic effects [102]. New vitrification methods requiring very small volumes and small concentrations of cryoprotectants and extreme cooling rates seem safer [103, 104]. However, new generation devices may require direct contact between samples and liquid nitrogen during vitrification with the risk due to the potential exposure to contaminants.

### Vitrified vs. slow frozen vs. fresh oocytes

Specific data on the influence of oocyte vitrification on obstetric risk are few and poorly reported, and head-to-head studies are lacking. A systematic review intercepted only uncontrolled or case reports reporting very limited data on the health of the newborns from vitrified and slow frozen oocytes. Also successive studies [105] reported reassuring pregnancy and neonatal data, even if the population studied were very little and poorly or not controlled. Only more recently, a retrospective study [106] on 954 pregnancies achieved after ET from slow frozen (n. 197) or fresh (n. 757) oocytes reported data on pregnancy and perinatal health. No differences were found between the use of fresh and frozen oocytes in the rates of ectopic pregnancies (3.6 % vs. 2.9 %) and spontaneous abortions (17.6 % vs. 26.9 %), and in gestational age at delivery [106]. However, the mean birth weights were significantly lower with fresh oocyte than with frozen-thawed oocytes, both in singleton (2.725 ± 727 g vs. 3.231 ± 615 g) and twins (2,128 ± 555 g 2,418- ± 92 g) [106]. Of interest, the analysis of the 63 patients who obtained pregnancies both in fresh and thawed cycles (138 pregnancies) showed no differences in the abortion rate and in the mean birth weight [106].

Only in 2014, a retrospective cohort study [107] compared the obstetric and perinatal outcomes of infertile patients who received IVF cycles after oocyte vitrification compared with those who received fresh oocytes. In particular, data from a total of 804 and 996 pregnancies, and from 1,027 and 1,224 children, respectively for vitrified and fresh oocyte group, were analyzed. No differences were found between the two techniques of cryopreservation in the rate of pregnancy complications, including obstetric, perinatal and puerperal problems [107]. In the vitrified oocytes group, the incidence of invasive procedures (aOR 2.12, 95 % CI 1.41 to 3.20), such as chorionic villous sampling or amniocentesis, and of urinary tract infections (aOR 0.51, 95 % CI 0.28 to 0.91) were respectively increased and reduced [107]. The proportion of female offspring was significantly higher in the vitrification group (53.8 % vs. 47.4 %) [107]. Main data did not change after sub-analysis for singleton and multiple pregnancies [107].

### Vitrified vs. slow frozen vs. fresh embryos

Comparable gestational age at delivery, weight at birth, and congenital anomaly rate were observed analyzing 435 blastocyst vitrification cycles using an open system, with the fresh oocyte control cycles [108]. Other researchers have confirmed those results in further studies after the vitrification of both cleavage stage embryos [109–112] and blastocysts [113, 114], or a combination of both [87]. One large study including 6,623 infants conceived after vitrification, found no difference also in terms of gestational perinatal mortality, although the incidence of SGA was lower and the weight at birth was heavier after vitrification in comparison with fresh ET [87]. Of note, all pregnancies and babies analyzed were conceived after SET.

One retrospective, single-center study of children born after day 3 ET from fresh, slow frozen or vitrified embryos showed that the incidence of PIH/PE, GDM, placenta previa and abruptio placenta were similar in all groups [112]. Specifically, no difference between vitrified and slow freezing day 3 embryos was observed in gestational ages at delivery, PTB, and perinatal mortality, even if the birth-weight of the babies born from vitrified embryos was higher in comparison with those of the babies born from slow frozen and fresh embryos, and the rate of LBW in vitrified embryos arm was significantly lower than in fresh embryos arm [112]. In twins, the birth-weight for children born from vitrified day 3 embryos was higher than that from the slow freezing, even if the difference cannot be considered clinically significant [112]. Unfortunately, a crucial recall bias cannot be excluded because the obstetric and perinatal data were obtained by questionnaires sent to the parents without checking medical records. Conversely, another recent retrospective study [113] demonstrated that the freezing methods of cleavage stage embryos did not exert any effect on the neonatal weight.

No difference in almost all pregnancy and perinatal/neonatal outcomes was observed in a Swedish retrospective study [114] comparing pregnancies achieved from vitrified blastocysts with those achieved from fresh blastocysts and slow frozen cleavage stage embryos. However, in pregnancies from vitrified blastocysts the incidence of SGA was lower (12.1 vs. 3 %) in comparison with fresh blastocyst, and the rate of major postpartum hemorrhage was higher (25 vs. 6 and 7.5 %) in comparison with fresh blastocyst and slow frozen cleavage stage embryos [114].

The largest and most recent population-based cohort study compared obstetric and perinatal outcomes of 16,845, 2,766 and 6,537 clinical pregnancies, and 13,049, 2,065 and 4,955 live deliveries of fresh, slow frozen and vitrified blastocysts [115]. Singletons born after transfer of vitrified but not slow frozen blastocysts had a lower risk of PTB (aRR 0.86, 95 % CI 0.77 to 0.96), LBW (aRR 0.67, 95 % CI 0.58 to 0.78) and SGA (aRR 0.60, 95 % CI 0.53 to 0.68) in comparison with singletons born after transfer of fresh blastocysts, [115]. The beneficial effect of vitrification on the birth weight of babies (and on the pregnancy duration) have been confirmed in a smaller retrospective study on 1,209 infertile patients who received a total of 1,157 fresh and 645 vitrified-warmed single blastocyst (day 5) transfers [116]. A higher infant birth weight has been demonstrated not only in singletons but also in twins after retrospective analysis of 784 fresh transfers and 382 vitrified-warmed DET at blastocyst stage [117].

An interesting retrospective study [118] analyzed the perinatal and neonatal health, adjusted for treatment variables and maternal characteristics, after 960 vitrified cycles and 1,644 fresh cycles according to the day of vitrification or fresh transfer, i.e. day 3 (cleavage stage) vs. day 5 (blastocyst stage). Singletons, but not twins, born after vitrification showed only lower SGA rate (aOR 0.55, 95 % CI 0.34 to 0.90) in comparison with peers born after fresh ET. The embryonic stage at vitrification or at fresh transfer did not influence any perinatal outcome [118]. These data seem to suggest that both vitrification and extensive culture at blastocyst stage can act and interact on fetal/neonatal weight.

### In vitro maturation (IVM) of oocytes

Any adverse effects of the IVM procedure on pregnancy and perinatal outcomes are a partially unknown field of research. From a mechanicistic point of view, IVM could affect the oocyte development, as any intervention in the growth phase could affect oocyte maturation, fertilization and subsequent embryo development [119]. On the other hand, the minimal gonadotropin stimulation, which very mildly stimulates the endometrium mimics natural environmental.

A systematic review [120] of head-to-head RCTs aimed to compare IVM followed by IVF/ICSI vs. conventional IVF/ICSI in terms of live birth rate and/or other efficacy and safety maternal/perinatal outcomes did not find any such study. Cha et al. [121] firstly reported on obstetric and perinatal outcomes in 41 IVM pregnancies suggesting no adverse effect of the technique. However, that data were uncontrolled and obtained in PCOS patients. Subsequent controlled studies confirmed the maternal and perinatal safety of IMV in comparison to IVF and/or ICSI, even if only few obstetric and perinatal data have been reported [122–124]. A slightly increased rate of GDM (17 % vs. 11 % and 10 % for IVM vs. IVF and ICSI, respectively) and of PE (12 % vs. 5 % and 8 % for IVM vs. IVF and ICSI, respectively) was observed in 55 IVM patients when compared with BMI and parity matched IVF/ICSI subjects (n. 217 and n. 160, respectively) [123]. No difference was observed all other pregnancy and perinatal outcomes among ART groups [123]. In singleton IVM pregnancies, the cesarean section (39 vs. 26 %) and instrumental delivery (9.5 vs. 6.5 %) rates were higher than in NC pregnancies but no difference between IVM and NC was detected in LBW (3 vs. 9 %), VLBW (0 vs. 2 %), gestational age (39 ± 3 vs. 39 ± 6 weeks), VPTB (0 vs. 2 %) [123]. Of interest, contrarily to IVF/ICSI neonates, the birth weight at delivery resulted heavier in IVM neonates (3,482 g vs. 3,260 g) than in NC neonates, and the PTB incidence (5 vs. 5 %) was not different from NC pregnancies [123]. A possible role of COH in determining a reduced birthweight in IVF/ICSI children has been also more recently suggested in a small but well controlled retrospective cohort study [125] performed on 196 IVM cycles compared with those of ICSI treatments. Although couples with a maternal age higher than 39 years or affected by azoospermia were excluded, the sample was heterogeneous as IVM cycles had different priming regimens and maturation techniques [125]. Only a difference in birth weight was observed between IVM and ICSI babies (3091 ± 669 g vs. 3269 ± 619 g) [125]. That difference may represent an inevitable influence of drug administration [125] but also an increased incidence of imprinting disorders [119, 126].

Although many studies on the safety of IVM are available in the literature, all have a retrospective design, very small sample sizes, and are not controlled and include a number of biases and (unadjusted) confounders. Obviously a high prevalence of PCOS cases in these studies could influence the results [11, 127]. In fact, a retrospective study [124] on 1,581 positive pregnancy tests demonstrated a rate of miscarriage after IVM significantly higher than after IVF/ICSI (25.3 % vs. 15.7 % and 12.6 %, respectively) but directly related to the PCOS rather than to the IVM procedure [124].

### Assisted hatching

Although hatching of the blastocyst is a crucial step for embryo implantation, and the failure to hatch can be considered one of limiting factors of the embryo development, the artificial disruption of the zona pellucida (assisted hatching) can potentially affect embryo competence and, thus, increase the risk of pregnancy complications. Although each technique used for assisted hatching can potentially exert specific effects on maternal and perinatal/neonatal health, very few data are available in literature about the effects of assisted hatching on obstetric and perinatal outcomes.

In a double-blind RCT [128], no effect on assisted hatching using acidic Tyroide’s solution was detected in women younger than 38 years on neonatal complications. Similarly, a retrospective cohort analysis [129] including a total of 699 women found no statistically significant effect of laser-assisted hatching, performed on day 3 in frozen (slow frozen and vitrified) embryos on gestational age, birth weight and Apgar score. The safety of the laser-assisted hatching was confirmed in singleton and twin pregnancies. Moreover, these data were limited by the heterogeneous subject selection due to inclusion criteria and patient choice.

Finally, the use of assisted hatching has been associated with an increased incidence of monozygotic twinning [130]. Moreover, available data are contrasting [101].

### Pre-implantation genetic diagnosis (PGD)

The European Society of Human Reproduction and Embryology (ESHRE) PGD Consortium reported that pregnancies and babies born after ICSI/PGD are not different from pregnancies obtained and babies born after ICSI treatment [131]. Similarly, gestational age, birth weight, and perinatal death were not different in a study analyzing 995 children born after ICSI/PGD and 1,507 children born after ICSI [132]. Interestingly, fewer multiples born after PGD presented a LBW [132]. These results, unfortunately, were not controlled for main confounders and did not have a NC group as reference.

A recent study [133] compared the perinatal outcomes of 245 neonates born after ICSI/PGD with those of neonates born to mothers matched for age, BMI and parity during the same period after ICSI (n. 242) and after NC (n. 733). The incidence of pregnancy complications after ICSI/PGD was low and not different from control groups whereas PTB and IUGR babies were more frequent in ICSI conceptions than in NC [133]. In singleton pregnancies, the weight of ICSI/PGD neonates, the rate of IUGR and LBW neonates and of PTB were not different from NC neonates but higher in comparison with ICSI neonates [133]. LGA was more frequent in the PGD group than after NC [133]. In twin pregnancies, no difference in LBW was detected between ICSI/PGD and NC twins, whereas significantly more ICSI twins presented with LBW [133]. That result did not change after sub-analysis for fresh and cryopreserved ET or for type of biopsy [133]. Of note, the mechanical partial zona dissection was the method for zona breaching in all cases of PGD.

A very recent retrospective cohort study, using data from the U.S. National ART Surveillance System on fresh autologous cycles with blastocyst transfer, compared 97,069 non-PGD procedures with other 9,833 PGD procedures demonstrating the influence on perinatal outcome of the specific indication for PGD [134]. Specifically, in women aged less than 35 years who had a live birth after ICSI/PGD the odd ratio of LBW infant was lower (aOR 0.73, 95 % CI 0.54 to 0.98) and higher (aOR 1.25, 95 % CI 1.01 to 1.54) in comparison with non-PGD cycles, when the indication was the detection of genetic disorders and aneuploidy screening, respectively [134]. On the other hand, no difference in any perinatal outcome was observed in women aged more than 35 years according to PGD indication [134]. Another recent multicenter-cohort study confirmed that the risk of adverse perinatal outcomes was mainly related to the underlying parental conditions rather than the PGD procedure [135]. In fact, after stratification of data for PGD indication, adverse obstetric and neonatal outcomes were only present in children conceived by PGD due to parental monogenetic disorder but not in children conceived after PGD due to parental chromosomal aberrations [135]. Nevertheless, there was a consistent risk of placenta previa (aOR 9.1, 95 % CI 3.4 to 24.9) after PGD and IVF/ICSI, suggesting that parental factors cannot explain all adverse outcomes [135].

Notwithstanding these available scientific data, definitive conclusions on the safety of the PGD cannot be drawn since couples undergoing PGD are usually fertile and in good health, data on fetal/perinatal growth and weight can be biased by timing of the biopsy, extended embryo culture, type of ET, etc., and long-term data on offspring are totally lacking. To this regard, a recent retrospective questionnaire analysis [136] showed a higher incidence of PIH (aOR 4.85, 95 % CI 1.34 to 17.56) in singleton pregnancies after blastocyst-stage biopsy and frozen ET than after cleavage-stage biopsy and fresh ET. In this regard, the Society of Obstetricians and Gynecologists of Canada [137] underlined that the data on an improved pregnancy outcome are inconsistent.

### Gamete and embryo donation

#### Oocyte donation (OD)

Initial studies on OD populations, aiming to assess their obstetric and perinatal risk, had as crucial confounders advanced maternal age and the high rate of multiple pregnancies and VTS [138–143]. In addition, they were based on relatively small cohorts, which did not allow subdivision into singletons and twins, with a higher twin rates in the OD group compared with the control groups [144–147].

An increased risk of obstetric complications, such as first trimester vaginal bleeding, PE, PIH, PTB and cesarean delivery, was reported after OD, although hypertensive disorders seem to be the most frequent one [138, 140–145, 148, 149]. With regard to perinatal outcomes, data are few and sparse, although most studies demonstrated no difference between OD and standard IVF [138, 144]. A large SART register study [150] indicated that children born after OD compared with IVF have lower birth weights and gestational ages at delivery. In particular, an increased risk of LBW (aOR 1.21, 95 % CI 1.13 to 1.30) and VLBW (aOR 1.28, 95 % CI 1.10 to 1.49) in OD singletons compared with IVF pregnancies was demonstrated [150]. Similar results were obtained previously in a cohort of 2,368 South American OD children [143]. Higher rates of LBW and PTB among OD singletons than in IVF/ICSI singletons was observed, although the perinatal mortality was similar [143].

Five meta-analyses on pregnancy complications and perinatal outcomes after OD cycles are available in the literature [151–155]. Unfortunately, these meta-analytic data were obtained without any adjustment for confounders and including studies with very high risk of biases. The first meta-analysis [151] was performed in order to confirm the high risk of severe hypertensive disorders observed in OD pregnancies in their daily clinical practice. After analysis of 28 studies, the odds for PIH/PE after oocyte donation was more than two-fold higher (OR 2.57, 95 % CI 1.91 to 3.47) in comparison with other infertility treatments, and more than six-fold higher in comparison with NC (OR 6.60, 95 % CI 4.55 to 9.57) [151]. The second meta-analysis [152] included 23 observational studies that compared the birth weight, mean gestational age at delivery and birth defects for conceptions after OD to those conceived from autologous oocytes [152]. Data were not controlled for confounders but analyzed only according to singleton or twin pregnancies. OD neonates were at an increased risk of PTB (RR 1.26, 95 % CI 1.23 to 1.30), LBW (RR 1.18, 95 % CI 1.14 to 1.22) and VLBW (RR 1.24, 95 % CI 1.15 to 1.35) when compared to autologous oocytes [152]. Very interesting data from sub-analysis demonstrated that in donor cycles the incidence of LBW was increased in PTB (RR 1.24, 95 % CI 1.19 to 1.29) but decreased (RR 0.86, 95 % CI 0.8 to 0.93) in term deliveries. The third meta-analysis included 11 observational studies for a total of 81,752 cycles comparing obstetric complications of pregnancies achieved after OD and autologous oocyte, and controlled for specific ART procedure (IVF or ICSI) [153]. In OD pregnancies the risk of developing PIH/PE, considered the primary endpoint, was significantly higher (OR 3.92, 95 % CI 3.21 to 4.78) also after sub-analysis for singleton (OR 2.90, 95 % CI 1.98 to 4.24) and twin (OR 3.69, 95 % CI 2.62 to 5.19) pregnancies [153]. Also the odds for SGA (OR 1.81, 95 % CI 1.26 to 2.60), PTB (OR 1.34, 95 % CI 1.08 to 1.66), and caesarean section (OR 2.71, 95 % CI 2.23 to 3.30) were increased [153]. Meta-regression for the covariate of age suggested that risk was independent of age [153]. Similarly, also the fourth meta-analysis [154] including 11 retrospective and prospective cohort studies confirmed that OD increase the risk of PE (in comparison with homologous IVF cycles) of about 70 % and that neither multiple pregnancies nor patient age can explain that effect by meta-regression analysis.

Finally, the last systematic review with meta-analysis [155] included, after search for original studies reporting at least five OD pregnancies with a control group of pregnancies conceived by conventional IVF/ICSI or NC and case series with > 500 cases, 35 studies reporting one or more pregnancy and perinatal complications were analyzed. The risk of PIH (aOR 2.30, 95 % CI 1.60 to 3.32), PE (aOR 2.11, 95 % CI 1.42 to 3.15), LBW (aOR 1.53, 95 % CI 1.16 to 2.01), PTB (aOR 1.75, 95 % CI 1.39 to 2.20) and CS (aORs 2.20, 95 % CI 1.85 to 2.60) was higher in OD than in IVF singleton pregnancies, whereas in multiple pregnancies only the incidence of PIH (aOR 2.45, 95 % CI 1.53 to 3.93) and PE (aOR 3.31, 95 % CI 1.61 to 6.80) was increased [155]. The risk of PE (aOR 2.94, 95 % CI 2.29 to 3.76), PTB (aOR 2.30, 95 % CI 1.09 to 4.87), LBW (aOR 1.94, 95 % CI 1.10 to 3.41) and CS (aOR 2.38, 95 % CI 2.01 to 2.81) was also increased in OD vs. NC singleton pregnancies [155]. Postpartum hemorrhage resulted increased in OD vs. IVF both in singleton (aOR 2.40, 95 % CI 1.49 to 3.88) and multiple (aOR 4.91, 95 % CI 1.22 to 19.83) pregnancies. No difference was detected in terms of GDM [155].

One of the largest and better controlled cohort study published on perinatal outcomes of children born after OD included 375 OD babies, and clinical data compared with three control cohorts of children, i.e. NC (33,852 babies matched by date and year of birth) and born after either IVF (11,060 singletons, and 6,532 twins) or ICSI (5,866 singletons, and 3,101 twins) [156]. An increased risk of PTB (aORs 1.8, 95 % CI 1.2 to 2.3; aOR 2.5, 95 % CI 1.7 to 3.6; and aOR 3.4, 95 % CI 2.3 to 4.9, respectively) and LBW (aOR 1.4, 95 % CI 0.9 to 2.2; aOR 1.8, 95 % CI 1.2 to 2.8; and aOR 2.6, 95 % CI 1.7 to 4.0, respectively) was detected in OD pregnancies vs. control pregnancies [156]. Of note, the risk of PE was also increased three-fold in OD pregnancies (aOR 2.9, 95 % CI 1.8 to 4.6; aOR 2.8, 95 % CI 1.7 to 4.5; and aOR 3.1, 95 % CI 1.9 to 4.9) [156]. The risk remained higher also after adjusting for maternal characteristics and after sub-analysis for twin pregnancies. Moreover, when the perinatal risk was adjusted for maternal PE the results improved, demonstrating no direct effect of OD on perinatal outcomes [156]. These data have been recently confirmed by a systematic review [157] showing that OD is an independent risk factor for PIH/PE, especially in twin pregnancies, and that its effect on fetal birthweight or growth is minimal after adjusting for obstetric complications. Finally, a very recent national registry study confirmed that OD recipients are more likely to have PTB (aOR 1.28, 95 % CI 1.12 to 1.46) and VPTB (aOR 1.30, 95 % CI 1.03 to 1.64) when compared with autologous patients, whereas the risk of having a SGA baby (aOR 0.72, 95 % CI 0.58 to 0.89) and of perinatal death (aOR 0.29, 95 % CI 0.09 to 0.94) was lower after adjusting data for gestational age [158].

#### Sperm donation

Sparse available data in the literature seems to suggest an increased risk of hypertensive disorders during pregnancy, with a specific increase of the PE risk, in nulliparous and in multiparous pregnancies with changed paternity [159, 160]. In this view, it could be hypothesized that the exposure to the paternal semen before conception has a protective effect, whereas the use of donor insemination after a previous pregnancy with paternal semen could increase the risk with an immune mechanism similar to hypertensive disorders seen in OD pregnancies. Unfortunately, at the moment data on sperm donation regard low technology interventions [11], and showed that the use of donor sperm in IUI cycles is associated with a risk of perinatal complications lower to those of the children born after partner sperm IUI and comparable to those of the NC children [44].

#### Embryo donation

Very little is known about the relationship between embryo donation and pregnancy and perinatal complications. In fact, available data can be extrapolated from infertile populations who conceived after mixed procedures of gamete and embryo donations [159]. At the moment, it very difficult to draw conclusions on the obstetric risk in women who had an embryo donation because the number of confounders and biases is so frequent and the detrimental effect of the double gamete donation can be only supposed. Commonly, the recipients are highly selected women with few medical comorbidities but who had had probably many previous ART failed attempts and a longer time-to-pregnancy. In addition, embryos donated are almost always frozen embryos. Finally, because embryo donation is more cost-effective than oocyte donation in case of male factor [161], the comparison in terms of pregnancy complication can be favorable for pregnancies obtained after embryo donation.

### Surrogate pregnancy

A systematic review on gestational surrogacy has been very recently published, and the pregnancy and perinatal outcomes of gestational carriers compared, when possible, with those of standard IVF and OD cycles [162]. The incidence of PIH ranged from 4.3 to 10 % and from 2.9 and 7.4 %, and the incidence of placenta previa and/or placental abruption from 1.1 to 7.9 % and from 1.1 to 3.7 %, respectively, in singleton and twin surrogate pregnancies [162], and resulted not different from those observed in IVF pregnancies and lower than that usually reported in OD pregnancies (ranged from 16 to 40 %) [163]. Three cases of hysterectomies related to delivery were also reported in gestational carriers and were due to uterine atony, placenta accreta and uterine rupture [162]. In surrogate singletons, the incidence of PTB and of LBW resulted, respectively, ranging from 0 to 11.5 % and from 0 to 11.1 % [162]. When compared to control groups, the risk of PTB and LBW was not different from IVF singletons (incidence of PTB of 14 % and of 13.6–14.0 %, respectively) and the risk of LBW was also not different from OD singletons (incidence of 14.0 %) [162]. However, a very recent US cohort study [164] underlined that the increased risk of PTB (aRR 1.14, 95 % CI 1.05 to 1.23) observed in gestational carriers is significantly influenced not only by multiple pregnancies but also by OD.

## Discussion

Despite the level and the quality of the current evidence it is generally suboptimal due to the presence of biases, confounders and limitations in study design (Table 3), current comprehensive review confirms that subfertile women who conceived after the use of high technology infertility treatments have an overall increased risk of pregnancy and perinatal complications, and highlights that every single step and/or procedure can play an independent and crucial role. In addition, several concomitant risk factors are frequently present in the same woman and influence the clinical and biological strategy of treatment. Thus, it is virtually impossible to define the weight of each reproductive treatment’s phase determining the whole patient’s risk. In fact, the infertility condition represents a bias per se in every study dealing with infertility treatments [11] and the presence of many confounding factors cannot be always adequately controlled through multivariate analysis because in many studies they are not clinically available, missing, or not collected.

**Table 3:** Specific biases, confounders and limitations present and/or declared in the available studies

Study	Design	Biases	Confounders	Limitations
Qin et al., 2016 [19]	Meta-analysis of 50 cohort studies	Little evidence of publication bias.	22 % of the included studies did not adjust and/or match any factors (i.e. maternal age, education, parity, race, occupation, smoking during pregnancy, socioeconomic status, etc.) when estimating the effect of ART singletons on obstetric outcomes.	- Patients who achieved a pregnancy with OI and IUI have been considered in the NC category. - Substantial heterogeneity among studies for association between ART singleton pregnancies and obstetric risks. - A number of the outcomes, especially for pregnancy-related complications, relied on between 2 and 7 of the 50 total studies.
Cromi et al., 2016 [21]	Case–control study	The study reports the experience of a tertiary referral center; therefore, this factor may have inflated the observed rates of peripartum hysterectomy.	The control populations have not been separated into two groups (normal fertile couples and infertile couples who conceive without treatment) to determine the degree to which observed associations are specifically related to the ART procedures vs. infertility per se.	- Detailed information on the infertility treatments was not available. - Small study number.
Pandey et al., 2012 [18]	Review of 20 matched cohort studies and 10 unmatched cohort studies	Ascertainment bias with the findings of increased complications with IVF/ICSI; i.e. women may be more anxious following fertility treatment and therefore more likely to report problems.	The quality of most of the studies was high and they have adjusted for most important confounders of age and parity.	- Some authors excluded pregnancies resulting from ovulation induction whereas others were not able to identify them from all non-IVF/ICSI conceptions. - The review cannot determine whether the increased risk is due to the inherent infertility itself or the process of ovarian stimulation and/or embryo culture.
Qin et al., 2015 [22]	Meta-analysis of 39 cohort studies	- No evidence of publication bias among studies of ART and risk of adverse outcomes. - All the included original studies used a cohort study design, which minimizes recall and selection biases.		- Some included studies have considered pregnancies arising after OI and IUI to be in the spontaneously generated category. - A number of the outcomes, especially for pregnancy-related complications, relied on between 1 and 8 of the 39 total studies. - The study population consisted of monochorionic and dichorionic twins, and monochorionic twins are known to be at high risk than dichorionic twins. - Residual confounding is a concern, although restricting analysis to studies that have matched or adjusted confounding factors did not materially alter the combined risk estimate.
Qin et al., 2016 [23]	Meta-analysis of 15 cohort studies	No evidence of publication bias.	26.7 % of the studies did not adjust and/or match any factors when estimating the effect of ART on obstetric outcomes in dichorionic twin pregnancies.	- More than half of the included studies had a small sample size. - Most of included studies belonged to retrospective cohort design. - There was substantial heterogeneity among studies for association between ART and obstetric risks in dichorionic twin pregnancies. - A number of the outcomes, especially for maternal complications, relied on between 2 and 7 of the 15 total studies.
Pinborg et al., 2013 [9]	Meta-analysis of 3 studies (for the considered outcome: PTB in SET vs. DET)	Subfertility per se is a bias and it cannot be prevented directly.	Vanishing twin pregnancies involve about 10 % of pregnancies with a DET-only strategy, leading to growth disturbances and to non-optimal perinatal outcomes among ART singletons.	
Marton et al., 2016 [35]	Longitudinal, retrospective cohort study	The focus was not on procedure specifics, even if each artificial procedure has profound effect on the splitting of the zygote, which represents a bias in the comparison of spontaneous and IVF–ICSI VT-pregnancies.		The relatively low incidence of VTS denotes the low power of the statistical analyses.
Nakashima et al., 2013 [43]	Retrospective study based on national registry	The effect of the subfertility has not been prevented.	The dataset had information on few confounders.	Detailed information on the infertility treatments was not available.
Sunkara et al., 2015 [49]	Prospective cohort study	The effect of the different gonadotropin dosages has not been excluded.	The dataset had no information on important confounders such as smoking, BMI and the medical history of women during pregnancy.	- The dataset did not allow specific identification of women with PCOS and its anonymized nature did not make it permissible to analyze one cycle per woman. - Individual women would have contributed to more than one cycle and outcome in the data set which means that the true sample size is unknown.
Kalra et al., 2012 [61]	Retrospective cohort study	To attempt to control selection bias, subanalyses were performed.	- Adjusted analyses were performed and included variables that were considered clinically important, because they are associated with adverse outcome. - Data not adjusted for gonadotropin dose.	
Mäkinen et al., 2013 [65]	Retrospective cross-sectional cohort study	The effect of the subfertility has not been prevented.	- The study was not adjusted for smoking and for gonadotropin doses. - Because of insufficient perinatal data, the authors were no able to control additional factors known to affect pregnancy outcome such as PIH, PE and GDM.	Lack of control of the duration of infertility.
Maheshwari et al., 2012 [81]	Systematic review and meta-analysis of 11 cohort studies	Patients who have had fresh cycle may be different from those who had frozen replacement cycles.	Data not adjusted for confounders such as age, smoking, parity, duration of infertility, and pre-existing medical illness due to varied design of the studies	- This review is limited to data from observational studies - There is inconsistency in definition of outcomes, such as antepartum hemorrhage, congenital anomalies, and perinatal mortality. In addition, not all outcomes have been reported by all studies. - There is clinical heterogeneity in terms of the population sampled, design of studies, method of freezing, and regimens in replacement cycles. - There is uncertainty as to whether method of thawing and protocol used (natural or hormonally mediated cycle) for replacement has any bearing on different obstetric and perinatal outcomes.
Ishihara et al., 2014 [89]	Retrospective study based on national registry	The different protocols and criteria for the use of frozen ET and blastocyst transfer potentially could bias the data.	The Japanese registry is cycle based with complete anonymity, therefore, it is impossible to know the detailed background of the patients who underwent ART, e.g., gravidity, parity, previous uterine surgery, etc.	- Wide variability of data compiled from almost 600 clinics that are different in size, location, and other characteristics. - Lack of a national registry of perinatal outcomes (incomplete data on maternal and neonatal outcomes for the final analysis).
Pinborg et al., 2014 [98]	National register-based controlled cohort study with meta-analysis	A bias is very unlikely as data coding was based on national registers	The data were not adjusted for confounding factors, such as maternal BMI and GDM.	The size of the frozen ET/fresh sibling cohort was limited.
Cobo et al., 2014 [107]	Retrospective cohort study	To avoid any selection bias, the study included the entire population of women from the two analyzed cohorts as were originally present in the Clinic.		- The study analyzed all the births for which there was notification, and not the whole series of IVF/ICSI pregnancies achieved in the Institution during the study period. - The conclusions are based on retrospective data that were partially obtained through medical questionnaires. - Information on pregnancy losses before 24 weeks is lacking (i.e., ectopic pregnancies, early and late miscarriages, and terminations of pregnancy due to fetal abnormalities). - The statistical power may be limited to detect a minor increase in the incidence of negative rare outcomes (i.e., major congenital malformations)
Li et al., 2014 [115]	A population-based cohort study	The effect of the subfertility has not been prevented.	The study used each treatment cycle as the unit of analysis where one woman could be included in both fresh and thaw cycles.	- Lack of information available on clinical-specific cryopreservation protocols and processes for slow freezing-thaw and vitrification-warm of blastocysts and the potential impact on outcomes. The lack of consistent cryopreservation protocols and comparison of embryo qualities might over-estimate the successful rate of vitrification and under-estimate the successful rate of slow freezing of blastocysts. - The data are observational and hence conclusions concerning the biological effects of vitrification and slow freezing cannot be drawn from our study
Buckett et al., 2007 [123]	Observational study	Risk of an ascertainment bias.	The database includes women with PCOS and the effect on birth weight may be a result of the inherent PCOS, rather than as a direct result of the treatment modality.	- Retrospective design. - Small sample size.
Jing et al., 2016 [136]	Retrospective cohort study	The effect of the subfertility has not been prevented.	To reduce the significant differences in genetic disorders, number of transferred embryos, methods of genetic testing, and vanishing twin between the two groups a logistic regression was applied in the study.	- Retrospective design. - Small sample size. - The study focused on obstetrics and neonatal out- comes and only included patients who were > 28 weeks pregnant.
Storgaard et al., 2016 [155]	Systematic review with meta-analysis of 22 cohort studies and 13 annual report of ASRM	The effect of the subfertility has not been tested.	- OD patients are very heterogeneous regarding age, inheritance and infertility history. - Oocyte donors also constitute a heterogeneous group. This affects pregnancy rates, but it is not known whether it influences obstetric and neonatal outcomes.	- To ensure reliable results only studies of high and medium quality were included in the meta-analyses (of the 21 included cohort studies comparing an OD group to a control group only two were of high quality and 11 were of medium quality). - Some outcomes were poorly defined, e.g. only three out of 11 studies included a strict definition of gestational diabetes
Salha et al., 1999 [159]	Retrospective cohort study	The effect of the subfertility has not been prevented.	To limit the confounding variables, women who conceived with donated gametes were compared to age- and parity-matched controls from similar demographic backgrounds who conceived with their own gametes.	- Retrospective design. - Small sample size.
Söderström-Anttila et al., 2016 [162]	Systematic review of observational studies (cohort studies and case-series)	Cohort studies, but not case series, were assessed for methodological quality, in terms of risk of bias.		- Lack of high quality studies. - Most studies have small sample size, lack of controls and a low response rate. - Gestational and traditional surrogacy was not always separated in the studies.

*ART* assisted reproductive technologies, *ASRM* American society of reproductive medicine, *BMI* body mass index, *DET* double embryo transfer, *ET* embryo transfer, *GDM* gestational diabetes mellitus, *ICSI* intracytoplasmic sperm injection, *IUI* intrauterine insemination, *IVF* in vitro fertilization, *NC* natural conception, *OD* oocyte donation, *OI* ovulation induction, *PE* preeclampsia, *PIH* pregnancy-induced hypertension, *SET* single embryo transfer, *VTS* vanishing twin syndrome

Ideally, the knowledge of the pathogenesis of the increased risk of pregnancy and perinatal complications in women who receive high technology infertility treatments and of the specific mechanisms of action could be crucial for preventing them. Unfortunately, few data are available regarding the biological explanations of that increased risk. Many mechanisms have been hypothesized and regard the alterations of the early placentation including not only alterations in endometrial receptivity, genetic and epigenetic mechanisms of implantation, invasion and growth of the trophoblast but also genetic and/or epigenetic alterations of oocyte/embryos due to biological manipulations (extended culture, culture media, techniques of cryopreservation, etc.), and immunological intolerance in case of OD because the fetal genome is allogenic to the carrier [11, 90, 127, 142, 165–167]. Thus, in the next future, still remains the need and an effort should be made to understand the reasons of these risks in order to minimize or prevent them.

However, from the clinical point of view, the priority is not to precisely estimate the amount of the obstetric risk but to recognize the presence of one or more risk factors (infertility and subfertility causes, patient’s characteristics and specific ARTs-associated risks), to correct those modifiable and to strictly follow the resulting pregnancies with appropriate prenatal cares. In fact, the delay in receiving prenatal care increased the PTB risk, while more-frequent use of prenatal care significantly improved the birth weight among pregnancies at high risk including subfertile women [168]. Recently, a large nationwide population-based study demonstrated that an adequate and intensive prenatal care reduces the risk of adverse pregnancy outcomes in women with history of infertility [169]. Specifically, less than six prenatal visits (compared with equal or more than six prenatal visits) and prenatal visits performed after the 12th week of gestation (compared with prenatal visits performed at or before the 12^th^ week of gestation) are related with a risk lower of VLBW neonates (aOR15.1, 95 % CI 8.8 to 25.8; aOR 2.1, 95 % CI 1.2 to 3.8; respectively), LBW neonates (aOR 2.1, 95 % CI 1.5 to 2.5; aOR 1.6, 95 % CI 1.3 to 1.9; respectively), and preterm birth (aOR 2.2, 95 % CI 1.9 to 2.6; aOR 1.1, 95 % CI 0.9 to 1.3; respectively) [169]. That data, however, were limited to singleton pregnancies.

At the moment, well established strategies to identify, follow and manage infertile patients and/or patients who have receive an infertility treatment are lacking and only few papers suggest potential strategies of management consisting in a generic close pregnancy monitoring and diagnostic testing [137, 157]. Physicians should assess the pregestational risk of infertile women before start any fertility enhancement treatment and discuss with the couples the increased risk for maternal and perinatal complications in a view of risk to benefit ratio and the potential alternatives, suggesting also to avoid any medical intervention in case of high-risk patients [137, 170], such as in case of women of very advanced reproductive age (> 55 years) or advanced reproductive age (> 45 years) with medical conditions [171].

## Conclusion

Subfertile women who conceived after the use of high technology infertility treatments are at increased risk of pregnancy complications, and every single/specific step and/or procedure can play an independent and crucial role. Thus, all infertile patients scheduled for high technology infertility treatments should be clearly informed of that increased obstetric and perinatal risk in case of pregnancy, regardless of multiple pregnancy. A careful preconceptional counselling aimed to optimize the general health status of the pre-pregnant women is needed (to stop smoking, reduce BMI in overweight/obese patients, and so on), identifying and treating modifiable reproductive disorders [11] and, finally, an effort should be made to optimize the infertility treatments (milder stimulation, OHSS prevention, elective SET) in order to prevent or reduce the risk of pregnancy complications in these infertile women. Finally, further large cohort prospective studies are required to clarify the contribution of each single factor on pregnancy and perinatal outcomes.

## Abbreviations

aOR: Adjusted odd ratio
aRR: Adjusted relative risk
ART: Assisted reproductive technology
ASRM: American society of reproductive medicine
BMI: Body mass index
CC: Clomiphene citrate
CI: Confidence interval
COH: Controlled ovarian hyperstimulation
DET: Double embryo transfer
E2: Estradiol
EPTB: Early-preterm birth
ESHRE: European society of human reproduction and embryology
ET: Embryo transfer
FSH: Follicle stimulating hormone
GnRH: Gonadotropin-releasing hormone
GRADE: Grading of recommendations assessment, development, and evaluation
hCG: Human chorionic gonadotropin
ICSI: Intracytoplasmic sperm injection
IUGR: Intrauterine growth restriction
IUI: Intrauterine insemination
IVF: In vitro fertilization
IVM: In vitro maturation
LBW: Low birth weight
LGA: Large-for-gestational age
NC: Natural conception
NICU: Neonatal intensive care unit
OCEM: Oxford centre for evidence-based medicine
OD: Oocyte donation
OGTT: Oral glucose tolerance test
OHSS: Ovarian hyperstimulation syndrome
OR: Odd ratio
PCOS: Polycystic ovarian syndrome
PE: Preeclampsia
PGD: Pre-implantation genetic diagnosis
PIH: Pregnancy-induced hypertension
PRISMA: Preferred reporting items for systematic reviews and meta-analyses
PTB: Preterm birth
RCTs: Randomized controlled trials
RR: Relative risk
SART: Society for assisted reproductive technology
SET: Single embryo transfer
SGA: Small for gestational age
UK: United Kingdom
VLBW: Very-low preterm birth
VTS: Vanishing twin syndrome
